# Microbial modification of host long-distance dispersal capacity

**DOI:** 10.1186/1741-7007-7-32

**Published:** 2009-06-19

**Authors:** Sara L Goodacre, Oliver Y Martin, Dries Bonte, Linda Hutchings, Chris Woolley, Kamal Ibrahim, CF George Thomas, Godfrey M Hewitt

**Affiliations:** 1 School of Biological Sciences, University of East Anglia, Norwich, UK; 2 Experimental Ecology, Institute for Integrative Biology, ETH Zürich, Zürich, Switzerland; 3 Department of Biology, Terrestrial Ecology Unit, University of Ghent, Ghent, Belgium; 4 Seale-Hayne Campus, University of Plymouth, Newton Abbot, UK; 5 Department of Zoology, University of Southern Illinois, Carbondale, IL, USA; 6 Current address : Institute of Genetics, University of Nottingham, UK

## Abstract

**Background:**

Dispersal plays a key role in shaping biological and ecological processes such as the distribution of spatially-structured populations or the pace and scale of invasion. Here we have studied the relationship between long-distance dispersal behaviour of a pest-controlling money spider, *Erigone atra*, and the distribution of maternally acquired endosymbionts within the wider meta-population. This spider persists in heterogeneous environments because of its ability to recolonise areas through active long-distance airborne dispersal using silk as a sail, in a process termed 'ballooning'.

**Results:**

We show that there is spatial heterogeneity in the prevalence of two maternally acquired endosymbiont infections within the wider *E. atra *meta-population and we demonstrate through several independent approaches a link between the presence of one of these endosymbionts, *Rickettsia*, and the tendency for long-distance movement.

**Conclusion:**

This novel finding that particular endosymbionts can influence host dispersal is of broad importance given the extremely widespread occurrence of similar bacteria within arthropod communities. A bacterial phenotype that limits dispersal has the potential not only to reduce gene flow and thus contribute to degrees of reproductive isolation within species, but also to influence species distribution and thus overall community composition.

## Background

Aeronautic dispersal comprises the main mode of long distance movement for many arthropods and is an important factor determining their distribution. Aerial dispersal in spiders is known as 'ballooning' [1] and is preceded by a stereotypical 'tiptoeing' posture, which is exclusively used for aerial dispersal (comprising leg stretching, abdomen raising and production of silk threads that are used as sails). It is estimated that ballooning spiders can move in excess of 30 km per day [2] but the increased risk of predation and of landing in unsuitable habitat [1] means that ballooning is constrained by factors such as restricted habitat affinity [3] and habitat fragmentation [4,5].

The money spider *Erigone atra *(Araneae: Linyphiidae, Blackwall 1833) is one of the most common aeronauts in Western Europe [1]. Its strong tendency to balloon is thought to account for its persistence in heterogeneous environments [6] and it is considered to be an important pest-controlling agent within agricultural settings [7,8]. Previous studies have showed that this species' aeronautic dispersal can be influenced by both heritable and environmental factors [9,10].

It has been shown that linyphiid spiders, such as *E. atra*, are often infected by maternally acquired bacterial endosymbiotic bacteria such as *Wolbachia, Rickettsia, Spiroplasma *[11] and *Cardinium *[12]. Such bacteria in other arthropods are observed to increase their own fitness through increasing the reproductive success of infected females [13,14]. This is achieved through a variety of mechanisms including male-killing, feminization of male embryos, the induction of parthenogenesis, or by causing cytoplasmic incompatibility. Beyond these effects, impacts on further key aspects of host reproduction such as mating behaviour and resulting consequences for sexual selection have also been proposed [15]. Given that changes in demography may greatly influence the evolution of dispersal behaviour within meta-populations [16] we have studied the effects of such endosymbiont infections on *E. atra *dispersal. All experiments were carried out as blind trials.

## Results

### Dispersal behaviour after antibiotic treatment to remove endosymbionts

We compared the dispersal behaviour of wild-caught individuals (males and females) in which we had manipulated endosymbiont infections through treatment with the antibiotics tetracycline (T) or penicillin-G (P). Individuals within a control group were treated with water (C). *Wolbachia, Rickettsia *and *Spiroplasma *are all sensitive to tetracycline; *Cardinium *is sensitive to both tetracycline and penicillin. All four types of endosymbiont were detected by polymerase chain reaction (PCR, see *Methods*) in our control (C) population (*n *= 27 females: *Cardinium *70%, *Rickettsia *44%, *Spiroplasma *11%, *Wolbachia *0%; *n *= 19 males: *Cardinium *58%, *Rickettsia *47%, *Spiroplasma *16%, *Wolbachia *5%).

Bacterial transmission rates from females to eggs were used as a measure of antibiotic treatment efficiency given that tetracycline is bacteriostatic and inactive bacteria might remain in antibiotic-treated adults. Lower transmission rates of *Rickettsia *and *Cardinium *from infected females to egg sacs following tetracycline treatment suggested that these bacteria had been successfully targeted in this sex (see *Methods*). The proportion of adult males infected with *Cardinium, Spiroplasma *and *Wolbachia *conform to those expected if treatment has been successful (% infected C > T and P > T) but the proportions carrying *Rickettsia *did not (% infected C < T and P < T), thus in the absence of an alternative method of assessment we cannot rule out the possibility that our treatment of males was less effective.

**Figure 1:**
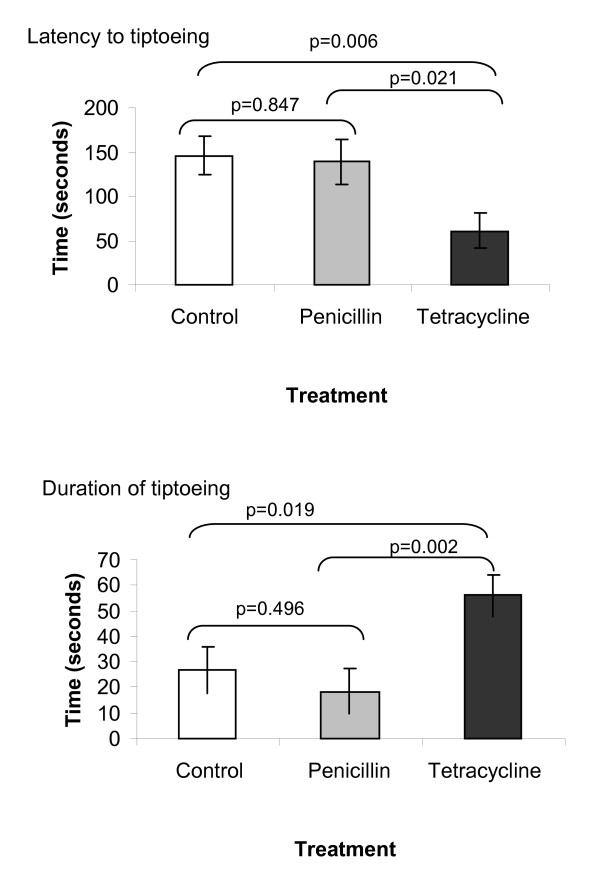
**Dispersal and antibiotic treatment**. Mean/S.E. of latency to exhibit ballooning behaviour and total time spent in ballooning posture (individual spiders observed for 6 minutes in total) of *Erigone atra* females subjected to one of three treatments: control/water (C), penicillin (P), tetracycline (T). P-values stem from post-hoc pairwise comparisons between treatments. Results for males (not on graph): mean tiptoe latency C 131.35 ± 25.59, P 28.22 ± 31.34, T 10.44 ± 27.14; tiptoe duration C 55.04 ± 11.12, P 86.54 ± 13.62, T 60.42 ± 11.80.

We compared the behaviour of T, P and C individuals in an artificial wind tunnel, designed specifically to simulate conditions suitable for dispersal. We used the adoption of a tiptoeing posture, which is exclusively associated with aeronautic dispersal and which is essential for becoming airborne, as our ballooning indicator. T females adopted this posture more readily than either C or P females (Figure 1). Multivariate GLM (MANOVA) analysis showed a significant effect of treatment on ballooning posture driven by significant effects on latency (*F*_2,48 _= 5.007; *P *= 0.011) and total time spent adopting the posture (*F*_2,48 _= 7.139; *P *= 0.002). Overall, significantly more T females (53%) adopted the ballooning posture than did those in C (26%, *χ*^2 ^= 5.81; *P *= 0.007) or P groups (23%, *χ*^2 ^= 5.81; *P *= 0.019). A similar analysis of male behaviour detected no significant effect of treatment on male ballooning posture. T and P group males had a lower latency (C *vs*. T *P *= 0.004, C *vs*. P *P *= 0.019) but the difference between T and P was not significant. There were no significant differences amongst treatment groups in the proportions of males exhibiting tiptoeing behaviour (C 26%, P 30%, T 42%, all *P *> 0.4).

Treatment with tetracycline is a widely employed method of curing arthropods of *Rickettsia *and *Wolbachia *infections but the potential side-effects of such a broad-spectrum antibiotic are not well characterised. We found no significant effect of treatment on the total number of eggs produced by females in our experiment (*F*_2,48 _= 0.138; *P *= 0.872) or on the manner in which they laid their egg sacs (females typically clump some of their egg sacs together but leave others individually spaced; the proportions of spaced egg sacs did not differ significantly amongst treatment groups, C 63%, P 74%, T 82%). We found no significant effect of treatment on general levels of activity, as measured by the amount of time individuals spent in a stationary position (*F*_2,93 _= 2.74; *P *= 0.07). Side effects of treatment seem unlikely to offer the full explanation for the observed difference in ballooning behaviour given that other traits such as reproductive output and levels of general mobility appear unchanged in response to the same treatment.

### Dispersal behaviour and endosymbiont infections in laboratory-reared animals

**Figure 2:**
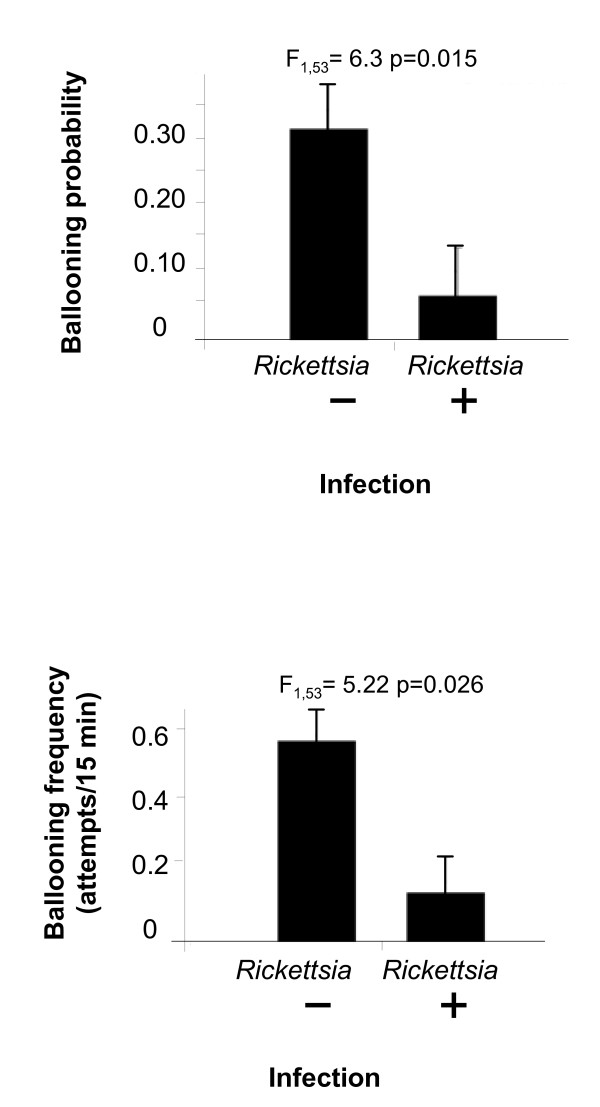
**Dispersal tendency and infection status**. Mean/S.E. of ballooning probability (upper) and ballooning frequency (lower) of individual *Erigone atra* females in relation to their *Rickettsia*-infection state (+/- infected/uninfected; assessed by PCR for *Rickettsia* citrate synthase gene).

We subsequently studied the lifetime reproductive success of captive-bred *E. atra *females and their behaviour under wind tunnel conditions (as in the experiment above) and then identified bacterial infections in these individuals by PCR [11]. *Rickettsia *(25%), *Spiroplasma *(6%), *Cardinium *(21%) but no *Wolbachia *were detected. We tested for associations between two types of dispersal behaviour that require silk to be produced: long-distance (ballooning) and short-distance (rigging, where anchored silk is used to swing the spider from one point to another close by). Given the results of our first experiment (where tetracycline but not penicillin treatment was associated with a behavioural difference) we tested for an association between *Rickettsia *infection and dispersal (Figure 2). *Rickettsia-*infected individuals had a reduced long-distance dispersal probability (*F*_1,53 _= 6.34; *P *= 0.015) and frequency (*F*_1,53 _= 5.22; *P *= 0.026). Other behaviours did not differ between *Rickettsia-*infected and uninfected females: probability of rigging, *F*_1,56 _= 0.09, *P *= 0.769; rigging frequency, *F*_1,53 _= 1.15, *P *= 0.337; rigging ability (= length of the produced silk thread), *F*_1,39.5 _= 0.34, *P *= 0.565; time spent web-building, *F*_1,52 _= 0.29, *P *= 0.59. No evidence was found that the infection state of mothers affected the sex-ratio of offspring (Wald *χ*^2 ^= 0.02; *df *= 12; *P *= 0.874) or their fecundity (*F*_1,12 _= 0.01; *P *= 0.931). *Rickettsia*-infected females, however, lived an average of 18.68 ± 8.45 days shorter than uninfected females (uninfected lifespan 50.98 ± 7.45 days; -36.64%; *F*_1,39.6 _= 4.89; *P *= 0.03). Similarly, the total number of egg sacs decreased from 7.45 ± 0.44 in uninfected to 5.14 ± 0.61 in infected females (*F*_1,52 _= 7.48; *P *= 0.0085) although we could detect no effect on overall lifetime fecundity (*F*_1,52.9 _= 2.11; *P *= 0.152). The latter is due to marginally lower numbers of eggs in the last egg sacs produced by uninfected individuals. Our data thus indicate a specific association between *Rickettsia *and dispersal, but only that of the long-distance kind. Subsequent tests established no similar effect of *Spiroplasma *or *Cardinium *on either type of dispersal movement.

### Dispersal behaviour and endosymbiont infections in natural populations

**Figure 3:**
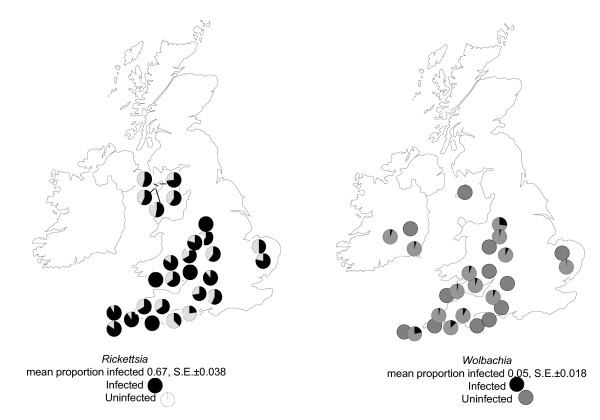
**Distribution of *Rickettsia* and *Wolbachia* in natural populations**. Location of sample sites and respective proportion of individuals found to be infected.

We studied levels of endosymbiont infections in a large number of individuals from a range of natural populations (Figure 3). All individuals were captured using d-vac sampling. We found several endosymbionts to be present (*Rickettsia *772 individuals tested, mean proportion infected 0.67, S.E. ± 0.038; *Wolbachia *796 individuals tested, mean proportion infected 0.05, S.E. ± 0.018). *Rickettsia *was found in a proportion of individuals from all 25 populations tested, but only 14 of these also carried *Wolbachia*. The mean proportion of individuals found to be infected with *Rickettsia *did not differ between populations where *Wolbachia *was also detected and those where it was not detected (mean proportion infected with *Rickettsia *where *Wolbachia *also detected = 0.65, S.E. ± 0.054; where *Wolbachia *not detected = 0.75, S.E. ± 0.046, *t *= 1.34, *df *= 25, *P *= 0.19).

We collected spiders from aerial [17] traps from two locations (wheat and grass fields respectively) near the Seale-Hayne Field Station, Devon, UK and compared endosymbiont infection frequencies in these spiders with those obtained from suction samples of the ground population at the same locations. Comparisons between aerial and ground samples must be interpreted carefully given the potentially larger action radius of aerial movers when compared with ground movers. Nonetheless, it is interesting that an analysis of the combined data indicates a significantly larger proportion of *Rickettsia-*infected females in ground samples (air 8%, ground 25%, exact test, *P *= 0.044). This is driven by a highly significant difference at the first site (air 11%, ground 83%, exact test, *P *= 0.002) with a larger (although not significantly so) proportion at the second site (air 7%, ground 15%, *P *= 0.32).

We detected no significant differences between aerial and ground samples for any of the other bacteria (*Wolbachia, Spiroplasma, Cardinium*) and no differences between aerial and ground males at either site for *Rickettsia *or any of the microbes. We observed that the distribution of individuals between aerial and ground samples varied significantly between the sexes. Males were almost twice as likely as females to be found in aerial traps (77% of the males at site 1 were found in aerial traps *vs*. only 43% of the females, *χ*^2 ^= 6.4, *P *= 0.011; at site 2 this was 72% of males *vs*. 29% of females, *χ*^2 ^= 30.16, *P *< 0.0001).

## Discussion

Our survey of natural *E. atra *populations demonstrates that endosymbionts, such as *Rickettsia*, are widespread but unevenly distributed within the spider meta-population as a whole. We find an increased tendency to disperse following antibiotic treatment and we show a negative relationship between *Rickettsia *and long-distance (but not short-distance) dispersal in the absence of antibiotic treatment. Together these different approaches provide strong evidence that long-distance dispersal of ballooning female spiders is influenced by specific microbial agents. Our observation that the proportion of individuals infected with *Rickettsia *differs between aerially dispersing and non-dispersing individuals trapped in their natural setting is consistent with these findings.

Few studies have directly examined the relationship between host dispersal rates and infection by parasites, despite the potential consequences for both the epidemiology of the infections themselves and the dynamics of the wider host meta-population [18]. As reviewed by Thomas *et al*. [19], altered host phenotypes may represent adaptations or side effects without any adaptive causality. Side effects that change host fitness may in themselves trigger subsequent selection. For instance, infections that lower host fecundity and maintain variability in host fitness theoretically lead to selection against long distance dispersal in spatially structured systems whilst not affecting short distance movements [20]. Dispersal strategies that are body-condition dependent are also shown to evolve under meta-population conditions with sufficient environmental stochasticity. In this instance individuals with the highest fecundity invest in dispersal while those with the lowest condition remain philopatric [21].

Both spatial structure and environmental stochasticity apply to the *E. atra *meta-population studied here and the arguments presented above appear to fit well with our observed data, particularly given that long-distance but not short-distance movement is found to be affected. However, reduced long-distance dispersal of female *E. atra *seems unlikely to be simply due to decreased host body condition and subsequent energetic constraints because we find no reduction in fitness associated with *Rickettsia *infection (although we cannot completely exclude the possibility that a hitherto undetected cost exists [22], in which case dispersal strategies could evolve in response to infection-dependent host fitness as proposed above). The fact that we also see no corresponding decrease in the tendency of infected individuals to use silk to move short distances, even though this may require the production of even more silk than during ballooning [10]), also indicates that there is no energetic constraint on dispersal. Nevertheless, as hypothesized above, selection might act differently on long- and short-distance dispersal strategies.

Another explanation is that reduced long-distance dispersal is an evolved adaptive modification by bacterial infections that promotes their own transmission. Endosymbionts are known to promote their own maintenance through mechanisms such as inducing cytoplasmic incompatibility (CI) or through altering the host sex ratio via parthenogenesis [23], male-killing [24] or feminization [25]. Further (theoretical) evidence indicates that reproductive parasites may also promote the maintenance of infection in a population through interfering in the dynamics of sexual conflict over mating rate, independently of effects on sex ratio or CI [15]. None of these phenotypes seems to occur in *E. atra *(all laboratory crosses of F1-females produce offspring; no sex ratio bias observed so far). However, changes in dispersal tendency could cause localised skews in the population sex ratio given that female behaviour appears to be influenced to a greater extent than that of males and that infection rates vary across the meta-population. Sex ratio distortions, even if subtle and transient, might impact on traits involved in interactions between the sexes [15,23].

Cytoplasmic incompatibility (CI) amongst different types/strains of geographically localized endosymbiotic bacteria has been hypothesized to explain reduced dispersal of spider mites where this increases the proportion of matings between individuals with the same infection type [26]. In this instance infected females were found to aggregate their offspring, thus encouraging sib matings (that is, between equally infected individuals). However, given that laboratory crosses give us no indication that there is CI in our study system, the above explanation seems unlikely to apply in this case. Furthermore, we found no similarly significant effects of endosymbionts on offspring aggregation (although we note that female spiders cured of their infections aggregated their egg sacs less than did the controls, fitting with the above rationale).

*Rickettsia *in *E. atra *appears to be associated with long-distance dispersal limitation in its host, potentially influencing gene flow and contributing to degrees of reproductive isolation and localised adaptation within the host meta-population, but without any reproductive modification. We have recently detected *Rickettsia *in the sub-oesophageal ganglion area of *E. atra *(the part of the nervous system where sensory and motor outputs are functionally linked), but not in the super-oesophageal ganglion (which is not directly involved in motor function), which may point to a potential mechanism through which the behavioural modification is achieved.

It remains unclear whether or not the behavioural change that we observed in female *E. atra *is a modification of host behaviour driven by selection for bacterial transmission. Future work will be directed towards investigating the interactions between host and bacteria in order to address this question. Laboratory populations of spiders can be cured with antibiotics and subjected to targeted re-infections (to eliminate effects of (or interactions with) other bacteria present). This will allow us to establish the fitness consequences for the host of carrying particular infections and to identify effects on other reproductive traits such as mating behaviour [27,28].

## Conclusion

We have demonstrated an endosymbiont-induced change in a non-reproductive phenotype that can impact on reproductive isolation via constraining gene flow and possibly altering local sex ratios. A recent estimate, and this just for one endosymbiont, *Wolbachia*, indicates that 66% of insect species may be infected with the bacterium, representing an impressive and considerable proportion of current worldwide biodiversity [29]. Although the impact of symbionts on reproductive traits has been the subject of intensive research, far less is known about effects on non-reproductive traits such as the reduced long-distance dispersal behaviour we present here. Our novel finding that the effects of maternally acquired endosymbionts on dispersal (and thus potentially on gene flow) can be an important force within a natural meta-population is thus of broad relevance. Finally, our findings also clearly demonstrate that the dynamics of ecosystem services such as a spider's pest-controlling function may be altered as a consequence of endosymbiont-driven shifts in host behaviour.

## Methods

### Molecular methods

Molecular tests for *Wolbachia, Rickettsia *and *Spiroplasma *in spiders and spider egg sacs were carried out using a variety of previously described PCRbased tests [11,12]. *Cardinium *was identified using the method of Zchori-Fein and Perlman [30].

### Antibiotic treatment and dispersal behaviour

In June 2005 we took 88 female and 58 male *E. atra *from a clover patch at Seale-Hayne Field Station, Devon, UK. We split spiders randomly into three groups and sprayed each group daily for 5 days with 0.1 ml 0.03% tetracycline (T), 0.03% penicillin-G (P) or distilled water (C). We note that spiders frequently groom themselves, a process that involves contact between the mouth and other body parts. This and drinking water droplets are considered likely routes for antibiotic ingestion. Effectiveness of treatment was assessed through studying bacterial transmission rates from infected females to egg sacs. Transmission of *Rickettsia *was significantly lower in the T group than in C (% infected females with infected egg sacs: C, 100 P, 100 T, 39, C *vs*. T *P *= 0.048, P *vs*. T *P *= 0.18, combined probability *χ*^2 ^= 9.50 *P *= 0.05). Transmission of *Cardinium *was also significantly lower in the T than C group with a (non-significant) decrease after penicillin treatment (% transmission C, 100 P, 50 T, 38, C *vs*. T *P *= 0.028, C *vs*. P *P *= 0.25). No females treated with tetracycline were found to carry *Spiroplasma *thus no comparison of transmission efficiency was possible for this bacterium.

Spiders were kept individually, fed 8 to 10 Collembola (days 1 and 3), left for 2 days then placed individually in a wind chamber (50 × 50 cm base with 8-cm vertical sticks 2.5 cm apart to provide launching platforms, 52 cm downwind of a fan, mean wind-speed 0.78 ms^-1^). The experiment was carried out twice per spider (two 3-minute trials on consecutive days). Sample sizes were: females C 27, P 28, T 33 (reduced to C 17, P 12, T 20 in the MANOVA because it included only females that tiptoed); males C 19, P 20, T 19 (reduced to C 9, P 11, T 10 in MANOVA). Egg counts were used as an independent measure of fitness for females but no comparable measure was available for males so data for the two sexes could not be combined in a single analysis. Dispersal behaviour of males and females was therefore analysed separately using a general linear model (GLM) in the software package SPSS.

### Protocols for testing dispersal behaviour of laboratory-reared spiders

Offspring from 13 randomly crossed F1-parents were individually reared on moist plaster of Paris at an optimal temperature of 25°C in petri dishes with a diameter of 4 cm. Until 1 week after maturation, spiders were fed *ad libitum *with *Sinella curviseta *(Collembola) and *Drosophila melanogaster *(Diptera). We placed a random selection of 57 females (taken exactly 1 week after their final moult) in a wind chamber [3] and recorded the frequency of short- and long-distance dispersal behaviours during 15-minute intervals. Both long-distance, aeronautic dispersal (tiptoeing followed by ballooning) and short distance dispersal (rigging) require silk and therefore constraints on silk production might influence both types of behaviour. Silk investment in web-building was assessed by placing individuals in specifically designed terraria with a grid of vertical structures (1 cm^2 ^grid size) to allow web-attachment and the size of the web to be subsequently measured [10]. Egg sacs produced by experimental individuals were tested for endosymbionts using molecular methods as described above.

None of the tested parameters (frequency of ballooning or rigging, duration of tiptoe behaviour, size of web) showed significant inter-correlations (all *R*_56 _< 0.23; all *P *= NS), thus we tested for associations between endosymbiont infections and long-distance and short-distance dispersal behaviour separately using Binomial mixed models (Proc Glimmix; SAS Institute Inc. 1999). We recorded fitness-related traits (female longevity after maturation and total clutch size) in order to infer possible condition-dependent dispersal. Models were corrected for potential similarity due to common origin (dam-effects) and for over dispersion. We used the Satterthwaite procedure to approximate denominator degrees of freedom. Similarly, fecundity and longevity were tested using mixed models with normal error structure (Proc Mixed; SAS Institute Inc. 1999).

### Dispersal behaviour and endosymbiont infections in natural populations

A survey was made of endosymbiont infections in spiders collected from ground suction samples at a range of locations (Figure 3). Samples were also collected from aerial [17] traps at two locations (wheat and grass fields respectively) near the Seale Hayne Field Station in Devon. First site: aerial trap: 24 males, 9 females; ground: 7 males, 12 females. Second site: aerial trap: 51 males, 27 females; ground: 20 males, 67 females. PCR test results for endosymbiotic bacteria in these individuals were: number of females infected/total in aerial traps (A) and from ground samples (G) for site 1 site 2: *Rickettsia *A: 1/9 2/27 G: 10/12 10/67;*Cardinium *A: 2/9 7/27 G: 3/12 24/67; *Spiroplasma *A: 1/9 2/27 G: 3/12 4/67. Males: *Rickettsia *A: 11/24 6/51 G: 2/7 5/20; *Cardinium *A: 4/24 22/51 G: 2/7 8/20; *Spiroplasma *A: 0/24 2/51 G: 0/7 0/20.

## Abbreviations

CI: cytoplasmic incompatibility; GLM: general linear model; PCR: polymerase chain reaction.

## Authors' contributions

SLG, OYM and DB were responsible for data handling and analysis and wrote the main text of the article. SLG did the molecular analyses, LH, CW and CFGT carried out the behavioural experiment with antibiotic treatment and collected specimens from ground and aerial traps. DB carried out the behavioural experiment with laboratory-reared spiders. KI and GMH contributed significantly to data analysis, interpretation and manuscript preparation.

## References

[B1] Weyman GS, Sunderland KD, Jepson PC (2002). A review of the evolution and mechanisms of ballooning by spiders inhabiting arable farmland. Ethol Ecol Evol.

[B2] Thomas CFG, Brain P, Jepson C (2003). Aerial activity of linyphiid spiders: modelling dispersal distances from meteorology and behaviour. J Appl Ecol.

[B3] Bonte D, Vandenbroecke N, Lens L, Maelfait J-P (2003). Low propensity for aerial dispersal in specialist spiders from fragmented landscapes. Proc R Soc Lond B Biol Sci.

[B4] Bonte D, Vanden Borre J, Lens L, Maelfait J-P (2006). Geographic variation in wolf spider dispersal behaviour is related to landscape structure. Anim Behav.

[B5] Bonte D, Bossuyt B, Lens L (2007). Aerial dispersal plasticity under different wind velocities in a salt marsh wolf spider. Behav Ecol.

[B6] Downie IS, Ribera I, McCracken DI, Wilson WL, Foster GN, Waterhouse A, Abernethy VJ, Murphy KJ (2000). Modelling populations of *Erigone atra *and *E. dentipalpis *(Araneae: Linyphiidae) across an agricultural gradient in Scotland. Agric Ecosyst Environ.

[B7] Sunderland K (1999). Mechanisms underlying the effects of spiders on pest populations. J Arachnol.

[B8] Madsen M, Terkildsen S, Toft S (2004). Microcosm studies on control of aphids by generalist arthropod predators: effects of alternative prey. BioControl.

[B9] Bonte D, Deblauwe I, Maelfait J-P (2003). Environmental and genetic background of tiptoe-initiating behaviour in the dwarf spider *Erigone atra*. Anim Behav.

[B10] Bonte D, Travis JMJ, De Clercq N, Zwertvaegher I, Lens L (2008). Thermal conditions during juvenile development affect adult dispersal in a spider. Proc Natl Acad Sci USA.

[B11] Goodacre SL, Martin OY, Thomas CFG, Hewitt GM (2006). *Wolbachia *and other endosymbiont infections in spiders. Mol Ecol.

[B12] Martin OY, Goodacre SL (2009). Widespread infections by the bacterial endosymbiont *Cardinium *in arachnids. J Arachnol.

[B13] Charlat S, Hurst GDD, Merçot H (2003). Evolutionary consequences of *Wolbachia *infections. Trends Genet.

[B14] Perlman SJ, Hunter MS, Zchori-Fein (2006). The emerging diversity of *Rickettsia*. Proc R Soc Lond B Biol Sci.

[B15] Hayashi TI, Marshall JL, Gavrilets S (2007). The dynamics of sexual conflict over mating rate with endosymbiont infection that affects reproductive phenotypes. J Evol Biol.

[B16] Bonte D, Hovestadt T, Poethke HJ (2008). Male-killing endosymbionts: influence of environmental conditions on persistence of host meta-population. BMC Evolutionary Biology.

[B17] Woolley C, Thomas CFG, Hutchings L, Goodacre SL, Hewitt GM, Brooks SP (2007). A novel trap to capture ballooning spiders. J Arachnol.

[B18] Boulinier T, McCoy K, Sorci G, Clobert J, Danchin E, Nichols JD, Dhondt AA (2001). Dispersal and parasitism. Dispersal.

[B19] Thomas F, Adamo S, Moore J (2005). Parasitic manipulation: where are we and where should we go?. Behav Processes.

[B20] Lion S, van Baalen M, Wildon WG (2006). The evolution of parasite manipulation of host dispersal. Proc R Soc Lond B Biol Sci.

[B21] Bonte D, de la Pena E (2009). Evolution of body condition-dependent dispersal in metapopulations. J Evol Biol.

[B22] Ims RA, Hjermann DO, Clobert J, Danchin E, Nichols JD, Dhondt AA (2001). Condition-dependent dispersal. Dispersal.

[B23] Hagimori T, Abe Y, Date S, Miura K (2006). The first finding of a *Rickettsia *bacterium associated with parthenogenesis induction among insects. Curr Microbiol.

[B24] Werren JH, Hurst GDD, Zhang W, Breeuwer J, Stouthammer R, Majerus ME (1994). Rickettsial relative associated with male killing in the ladybird beetle (*Adalia bipunctata*). J Bacteriol.

[B25] Hurst GDD, Werren JH (2001). The role of selfish genetic elements in eukaryotic evolution. Nat Rev Genet.

[B26] Vala F, Egas M, Breeuwer JAJ, Sabelis MW (2004). *Wolbachia *affects oviposition and mating behaviour of its spider mite host. J Evol Biol.

[B27] Champion de Crespigny FE, Wedell N (2006). *Wolbachia *infection reduces sperm competitive ability in an insect. Proc R Soc Lond B Biol Sci.

[B28] Champion de Crespigny FE, Pitt TD, Wedell N (2006). Increased male mating rate in *Drosophila *is associated with *Wolbachia *infection. J Evol Biol.

[B29] Hilgenboecker K, Hammerstein P, Schlattmann P, Telschow A, Werren JH (2008). How many species are infected with *Wolbachia*? A statistical analysis of current data. FEMS Microbiol Lett.

[B30] Zchori-Fein E, Perlman SJ (2004). Distribution of the bacterial symbiont *Cardinium *in arthropods. Mol Ecol.

